# Respiratory support after extubation: noninvasive ventilation or high-flow nasal cannula, as appropriate

**DOI:** 10.1186/s13613-017-0271-8

**Published:** 2017-05-18

**Authors:** Tommaso Mauri, Giacomo Grasselli, Samir Jaber

**Affiliations:** 1 Department of Pathophysiology and Transplants, University of Milan, Milan, Italy; 2 Fondazione IRCCS Ca’ Granda Ospedale Maggiore Policlinico, Milan, Italy; 3 Department of Anesthesiology and Critical Care Medicine B (DAR B), Saint-Eloi Hospital, University Teaching Hospital of Montpellier, INSERM U1046, 80 Avenue Augustin Fliche, 34295 Montpellier, France

## Editorial

Decreasing the duration of invasive mechanical ventilation by early safe extubation is a major clinical goal in intensive care unit (ICU) [1]. Prolonged intubation increases the risk of ventilator-induced lung injury, ventilator-induced diaphragm dysfunction, myopathy and infections. Nonetheless, patients’ management in the post-extubation period can be challenging and every effort should be made to avoid re-intubation, which is associated with significantly increased morbidity and mortality [1].

To this end, in this issue of the “Annals of Intensive Care,” Dr. Fernandez and colleagues report findings from a randomized controlled trial comparing low-flow oxygen supplied through nasal prongs or facial mask versus high-flow nasal cannula (HFNC) for 24 h after extubation as respiratory supports in patients at high risk of extubation failure [2]. This is the third large clinical trial recently published by the research group of Dr. Fernandez on the same topic, the other two being comparisons of HFNC versus noninvasive ventilation (NIV) in high-risk patients and of HFNC versus standard oxygen in low risk [3, 4]. In this issue study [2], patients’ population consisted of adult critically ill patients receiving mechanical ventilation for more than 12 h, considered as high risk of extubation failure and extubated after successful spontaneous breathing trial (SBT). High risk of extubation failure was defined as the presence of at least one among the following: age above 65 years old, heart failure, moderate or severe chronic obstructive pulmonary disease, APACHE II score higher than 12, body mass index above 30 kg/m^2^, weak cough with abundant secretions, more than one SBT failure and/or mechanical ventilation for more than 7 days. The primary study outcome was the development of respiratory failure within 72 h after extubation, with an expected incidence of 28% in the conventional oxygen group versus 21% in the HFNC group. Re-intubation, length of stay and mortality were secondary outcomes.

We must underline that the study has several limitations, in part acknowledged by the authors. First, avoiding use of NIV in high-risk patients might pose serious ethical issues as it might be failure to apply a treatment that was clearly shown to improve clinical outcome [5]. Second, the planned sample size for the primary outcome was 1184 patients (592 per arm), but the study was stopped at 155 due to slow recruitment (average of 1.6 patients per unit per month). Third, it is questionable whether assessment of the risk of extubation failure should be performed beforehand without any post-extubation reassessment: For example, a patient with early signs of respiratory failure (e.g., desaturation within 1 h from extubation) but no pre-defined risk factors might be regarded as more at risk than a patient presenting only some pre-extubation risk factors. Fourth, HFNC was arbitrarily implemented for 24 h only, while in everyday clinical practice, its discontinuation would more likely be based on the patient’s clinical evolution. Fifth, no physiologic test was performed to guide randomization (i.e., predictive enrichment: HFNC could have been implemented only in patients who have decreased respiratory rate after 30 min of treatment). Finally, patients developing hypercapnia during the SBT were excluded; thus, findings from this study do not apply to this population, which might be the most clinically relevant [5] and should be considered as post-extubation NIV application. Despite these limitations, we must recognize that study findings on the role of HFNC in the post-extubation period are relevant and encouraging: Incidence of respiratory failure reflected the hypothesized reduction granted by HFNC and re-intubation somehow decreased, too. Mortality and length of stay were low and similar in both groups. Moreover, the authors performed a multivariate logistic regression analysis to identify factors independently associated with post-extubation respiratory failure. Candidate variables were: HFNC, diagnosis of cancer, days on mechanical ventilation before enrollment, diagnosis of chronic obstructive pulmonary disease (COPD), body mass index (BMI) and heart failure as etiology of respiratory failure. The only factor independently associated with prevention of respiratory failure was HFNC, while diagnosis of cancer increased the risk.

After the switch from invasive mechanical ventilation to unassisted spontaneous breathing, the following untoward effects may arise as direct consequence of the sudden loss of positive intrathoracic pressure [6]: End-expiratory transpulmonary pressure decreases to a level that might be below the closing volume of relatively unstable alveoli, posing risk of collapse and atelectasis; the difference between intravascular capillary pressure and alveolar pressure increases, both directly and as a result of increase in cardiac output, increasing the risk of alveolar transudation and edema. Moreover, after removal of the endotracheal tube, the airway lumen diameter might abruptly decrease, especially during inspiratory efforts, increasing airway resistance and, at high airflow rates, further decreasing alveolar pressure and increasing the work of breathing. These phenomena might initiate a vicious circle that leads to worsened oxygenation and increased hypoxic drive, inspiratory effort and consequently transpulmonary pressure. Heterogeneity of the lung parenchyma due to atelectasis could lead to extremely elevated regional driving transpulmonary pressure, further worsening lung injury and edema. The physiologic effects of HFNC might perfectly match the patient need during the post-extubation period [7–9]. HFNC generates a “positive end-expiratory pressure (PEEP) effect” that might increase end-expiratory transpulmonary pressure stabilizing the alveoli, increase alveolar pressure decreasing the hydrostatic capillary-alveolar gradient and increase the airways caliber. Moreover, HFNC improves oxygenation and CO_2_ clearance, thus decreasing the hypoxic and hypercapnic drives and the inspiratory effort. In this way, driving transpulmonary pressure decreases, preventing further injury, and work of breathing is reduced, preventing fatigue [10]. Finally, heated humidified gas delivered by HFNC can promote secretions fluidity and clearance. These data suggest that HFNC could effectively contribute to interrupt the post-extubation vicious circle of edema, excessive effort, lung injury and muscle fatigue, facilitating full recovery of lung function.

In conclusion, as described by the study of Dr. Fernandez and colleagues [2], HFNC might be a powerful respiratory support in the post-extubation period with multiple physiologic and clinical benefits. However, use of HFNC should be limited to patients at risk of re-intubation, while NIV should be considered promptly in COPD patients and in those with rapid deterioration of the respiratory function while on HFNC.

## Abbreviations

BMI: body mass index
COPD: chronic obstructive pulmonary disease
HFNC: high-flow nasal cannula
ICU: intensive care unit
NIV: noninvasive ventilation
PEEP: positive end-expiratory pressure
SBT: spontaneous breathing trial
